# The first report from Ankara on the presence of xylazine abuse in blood and urine samples using a validated LC-HRMS method

**DOI:** 10.1007/s00414-025-03562-7

**Published:** 2025-07-05

**Authors:** Yeter Erol Öztürk, Oya Yeter, Hızır Aslıyüksek, Ali Oztuna, Sermet Sezigen, Göksun Demirel

**Affiliations:** 1 Council of Forensic Medicine, Chemistry Department, Ankara, 06300 Turkey; 2 Council of Forensic Medicine, Chemistry Department, Istanbul, 34196 Turkey; 3 Council of Forensic Medicine, Morgue Department, Istanbul, 34196 Turkey; 4 Gulhane Training and Research Hospital, Ankara, 06010 Turkey; 5 Dept. of Medical CBRN Defense, University of Health Sciences, Ankara, 06010 Turkey; 6 Faculty of Pharmacy, Department of Pharmaceutical Toxicology, Cukurova University, Adana, 01380 Turkey

**Keywords:** Xylazine, Abuse, Adulterant, Blood, Urine, LC-HRMS

## Abstract

Xylazine, an alpha-2 adrenergic receptor agonist approved for veterinary use, has emerged as a significant adulterant in illicit drugs, particularly synthetic opioids like fentanyl, contributing to a rise in fatal overdoses. Despite its widespread abuse in the United States and other regions, data on xylazine prevalence in Türkiye remain limited. This study aimed to develop and validate a sensitive and rapid liquid chromatography high-resolution mass spectrometry (LC-HRMS) method for detecting xylazine and its metabolites, 2,6-dimethylaniline (DMA) and 4-hydroxyxylazine, in human blood and urine samples. Solid-phase extraction (SPE) was used for sample preparation and the method demonstrated high sensitivity with limits of detection of 0.025–0.06 and 0.068 0.19 ng/mL for xylazine and its metabolites in blood and urine, respectively. Between January and December 2024, 9,123 biological samples from suspected drug users in Ankara were analyzed for xylazine and its metabolites. Xylazine was detected in 28 cases (0.3%). While 4-hydroxyxylazine was detected in 78% of positive cases, DMA was detected in 25% of positive cases. Polysubstance use was common, with pregabalin (50%) and methamphetamine (28%) being the most frequently co-detected substances. This study represents the first report of xylazine abuse in Türkiye, highlighting its emergence in the local drug supply. The findings underscore the need for routine xylazine screening in forensic toxicology to monitor its prevalence and mitigate its impact as an emerging public health threat. The developed LC-HRMS method offers a rapid, sensitive, and reliable tool for detecting xylazine and its metabolites, supporting efforts to address this growing concern.

## Introduction

Xylazine (C12H16N2S) is a partial presynaptic alpha-2 adrenergic receptor agonist and it was approved by the United States Food and Drug Administration (FDA) in 1972 for large animals in veterinary practice. It causes sedation, muscle relaxation, analgesia, and euphoria by decreasing the release of norepinephrine at presynaptic level and dopamine from the central nervous system (CNS). Inhibiting norepinephrine transport competitively reduces sympathetic outflow and causes bradycardia, hypotension, respiratory and CNS depression which are also the symptoms of xylazine-involved overdose cases [1–11]. Initially, xylazine was developed as an antihypertensive agent due to its similarity to clonidine by Farbenfabriken Bayer, Germany, in 1962. However, lethal adverse effects in humans, including bradycardia, severe hypotension, transient hyperglycemia, respiratory depression, and CNS depression, prevented its initial approval by the FDA [12–15]. Xylazine could be legally supplied with a veterinary prescription all over the world as it is not listed as a controlled substance. Due to its increased availability, it is widely used as an emerging drug of interest in various street drugs including fentanyl and heroin across the United States. Since fentanyl became the leading illicit drug in the United States in 2016, the use of xylazine with synthetic opioids like fentanyl has increased the risk of fatal overdoses due to their synergistic effects [16]– [17].

Xylazine abuse was first documented in Puerto Rico in 2001 where it is also called “anestesia de caballo” (horse anesthetic), and in Philadelphia in 2006 where it is popularly known as “tranq”. As an attractive cost-cutting adulterant using in common practice, it is often mixed with some illicit substances including heroin, fentanyl, cocaine, and methamphetamine for increasing the bulk of the product by decreasing the amount of illicit substance [4]. Xylazine was noted as a common additive in the Philadelphia opioid supply since mid-2010’s [1]. Although the addition of adulterant substance into the primary illicit drug increases the profit of drug dealers, this action causes the interference of pharmacological effects and increases the potency of polydrug [11]. For this reason, misdiagnosis of polydrug overdose cases involving xylazine could be fatal due to improper lifesaving interventions. It was reported that xylazine was also used with morphine, codeine, and alcohol. While xylazine extends the duration of analgesia and euphoria of induced opioids like fentanyl which is a short acting one, and potentiates the effects of the drug, it decreases the adverse effects and withdrawal symptoms of the stimulants. However, xylazine abuse itself causes severe withdrawal symptoms, including anxiety, dysphoria, and irritability, for which there are currently no treatment options. “Tranq-dope” that xylazine is mixed with fentanyl or heroin reduces the re-dose frequency of the drug by extending the desired period of euphoria and sedation. This made tranq-dope popular in the illicit drug markets of the eastern states of the Unites States and most recently in Canada. In Puerto Rico, xylazine is also mixed with “*speedball”* which includes heroin and cocaine for balancing the “down” effect of heroin and xylazine [1, 3, 4, 6–8, 10, 12, 15, 18, 19]. Xylazine has a nickname of “*zombie drug*” as it causes chronic skin degradations. Intravenous, inhalation, intramuscular, and subcutaneous administrations are common routes for xylazine use, respectively [6, 7, 10, 12, 13, 20]. However, intravenous administration is more prevalent among xylazine users in North America [17]. The literature also reported accidental or intentional exposures to xylazine [21].

Typical xylazine overdose symptoms including bradycardia, hypotension, hypothermia, hyperglycemia, “high” feeling, dry mouth, miosis, and coma due to increased brain hypoxia could be seen between 15 min (min) and 2 h (h) after the exposure. The duration of overdose could be up to 72 h. The concomitant use of xylazine with opioids could increase the incidence of fatal overdose by potentiating the current symptoms and the respiratory depressant effects of opioids. However, differential diagnosis of xylazine seems to be problematic because these cases are mostly admitted to the emergency departments with opioid overdose use and most of the physicians have limited awareness about overdoses involving xylazine. Additionally, point of care tests for xylazine and fentanyl are not available in most of the hospitals in the United States [5, 7, 10, 12, 15, 19]. For this reason, there is an increased risk of delayed diagnosis and inadequate medical management for the cases with polydrug overdose including xylazine and fentanyl because of the absence of established guidelines and protocols which are specific for the treatment of xylazine overdose [6, 16].

Long term use of xylazine, which is also a potent vasoconstrictor, causes injection-related soft tissue infections, severe necrotic skin ulcers and necrosis at injection sites, which may cause pain, immobility and even extremity amputations. The pathophysiological mechanism of the injury is still being investigated especially from the perspective of vascular endothelial damage [6, 9, 14, 18].

It was reported that of polysubstance use involving xylazine caused 80% of opioid overdoses in the United States. Xylazine both increases the lethal effects of fentanyl, makes the lethal overdoses more common, and complicates the antidotal management of opioid overdose cases [16]. It was reported that xylazine was a cause of death in 64.3% of fatal overdose cases in the United States in 2019 [12]. CDC indicated an 276% increase in the identifying of xylazine in fentanyl overdose deaths between 2019 and 2022 in the United States [3]. FDA warned healthcare authorities on November 8, 2022, with a statement about the increased prevalence of xylazine-involved opioid (fentanyl) overdose cases because it was reported that fatal drug overdose with xylazine increased 20 times between 2015 and 2020 in the United States [9]. CDC estimated that there were 74.702 overdose death occurring synthetic opioids, primarily fentanyl in 2023 and the number of these deaths are more than the annual number of deaths from motor vehicle and firearm accidents [16, 22]. As March 2023, law enforcement forces found xylazine in illicit fentanyl supplies in 48 states of the United States. On April 12, 2023, xylazine combined with fentanyl was declared by the United States federal government as a designated widespread emerging threat that causes numerous overdose death [4–7]. According to the 2024 UNODC publication Current NPS Threats Volume VII, xylazine was detected in 123 cases of available post-mortem (PM) data, 87% of which also included fentanyl and 49% methamphetamine. All of the 16 xylazine-related clinical cases were associated with polydrug use. Patients typically consumed a combination of various other new psychoactive substances (NPS) and illicit substances, including fentanyl, exhibiting symptoms of overdose [23].

Xylazine abuse is associated with increased public health concerns including overdose deaths due to respiratory depression, suicide, human immunodeficiency virus, and hepatitis C virus infections [1, 2]. Prolonged loss of consciousness and atypical withdrawal syndromes make it popular in drug-facilitated crimes including sexual assault, robbery, and homicidal intent [2, 5, 10, 12].

There is limited data in the literature about the human metabolic pathways of xylazine. Because of its lipophilic character, xylazine has a large volume of distribution and it is rapidly eliminated from the human body. Besides, it quickly crosses the blood-brain barrier, and its sedative effects become more potent. The plasma half-life of xylazine was reported as 4.4 h. In the liver, it is metabolized by cytochrome P450 enzymes. Hydroxylation, oxidation, N-dealkylation, and S-oxidation are phase I metabolic reactions for xylazine in humans. Xylazine is demethylated by hepatic enzymes into 2,6-dimethylaniline (DMA) which is the major metabolite of xylazine. Xylazine and its phase I metabolites can be conjugated with glucuronic acid or sulfate, forming hydroxylated metabolites including 4-hydroxyxylazine, and they are excreted into urine through phase II metabolic reactions. Recent studies showed that approximately 70% of xylazine is excreted intact into human urine [5, 6, 10–12, 14, 15, 20, 24–27].

Drug abusers in the United States, Canada, and western countries preferred xylazine as an adulterant over a decade ago and the growing spread of its illegal use increases the risk of overdose and death around the world. For this reason, the prevalence of xylazine use in the illicit drug market should be monitored to understand and to mitigate the effects of this emergent threat [17]. Due to lack of xylazine testing in routine toxicological analysis pattern as xylazine is a veterinary pharmaceutical drug, there is no data about accurate case number of xylazine abuse in Türkiye. For this reason, we added xylazine and its metabolites to the general toxicological screening panel of human biological samples including blood and urine in our Ankara laboratory on January 01, 2024. The aims of this study were to develop and to validate a sensitive and fast LC-HRMS method for the quantitation of xylazine and main metabolites in human blood and urine samples after the solid phase extraction. In this context, we also aimed to detect the prevalence of xylazine in biological samples of suspected illicit drug users in Ankara during 2024 for a better understanding of xylazine abuse risk. Our study is the first report from Türkiye, and we also believe that sharing information is important for supporting effective early warning systems on drugs.

## Materials and methods

### Materials

Xylazine, 2,6-dimethylaniline (DMA), and 4-hydroxyxylazine were purchased from Chiron (Trondheim, Norway). Xylazine-d6 as internal standard (IS) was purchased from Cayman Chemical (Ann Arbor, MI, USA). The purity of the chemicals was greater than 98%. LC-MS grade solvents including acetonitrile, ultrapure water, and formic acid were obtained from VWR (Vienna, Austria). SPE cartridges (Oasis HLB, 60 mg, 3 mL) were purchased from Waters (Milford, USA). Stock solutions of xylazine, DMA, 4-hydroxyxylazine, and xylazine-d6 were prepared by dissolving the chemicals in methanol. After preparation, stock solutions (0.1 mg/mL) were stored at −20 ℃ in glass vials until use and diluted with methanol.

This study was approved by the Presidency of the Scientific Board of the Council of Forensic Medicine (21589509/2024/691). Biological samples including blood and urine were provided by suspects under the supervision of the judicial authorities for drugs of abuse testing with official documentation. Whole blood and urine samples were collected and identified according to the sampling procedures of the Council of Forensic Medicines in Ankara and stored at −20 ℃ until analysis.

### Sample preparation

2.5 mL water was added to 500 µL sample (whole blood or urine). Then 50 µL IS (50 ng/mL) was added to each sample. The sample was centrifuged for 5 min at 4.000 rpm. SPE cartridges were conditioned with 2 mL methanol and then equilibrated with 2 mL ultrapure water. 0.5 mL of sample was passed through the cartridge. The cartridge was washed with 2 mL of a 5% methanol solution in ultrapure water to remove impurities. The cartridge was then dried under a gentle positive airflow for 10 min. The sample was eluted with 2 mL of methanol. The eluate was evaporated to dryness under a gentle nitrogen flow and reconstituted with 0.5 mL of ultrapure water: acetonitrile (85:15; v: v). Then, it was injected to LC-HRMS.

### LC-HRMS analysis

Liquid chromatography was performed on a Vanquish system which was equipped with a UPLC BEH C18 column (130 Å, 2.1 mm x 100 mm, 1.7 μm) from Waters (Milford, USA). The column temperature was set at 40 °C. Mobile phase A consisted of 0.1% formic acid in ultrapure water and mobile phase B consisted of 0.1% formic acid in acetonitrile. The step gradient was as follows; 15% mobile phase B at t = 0 min, linear gradient to 90% B at t = 3 min, kept at 90% B till t = 5 min, linear gradient to 15% B and then equilibrated to initial conditions at t = 7 min. The total running time was 7 min. The flow rate was 600 µL/min, and the injection volume was 10 µL.

**Fig. 1:**
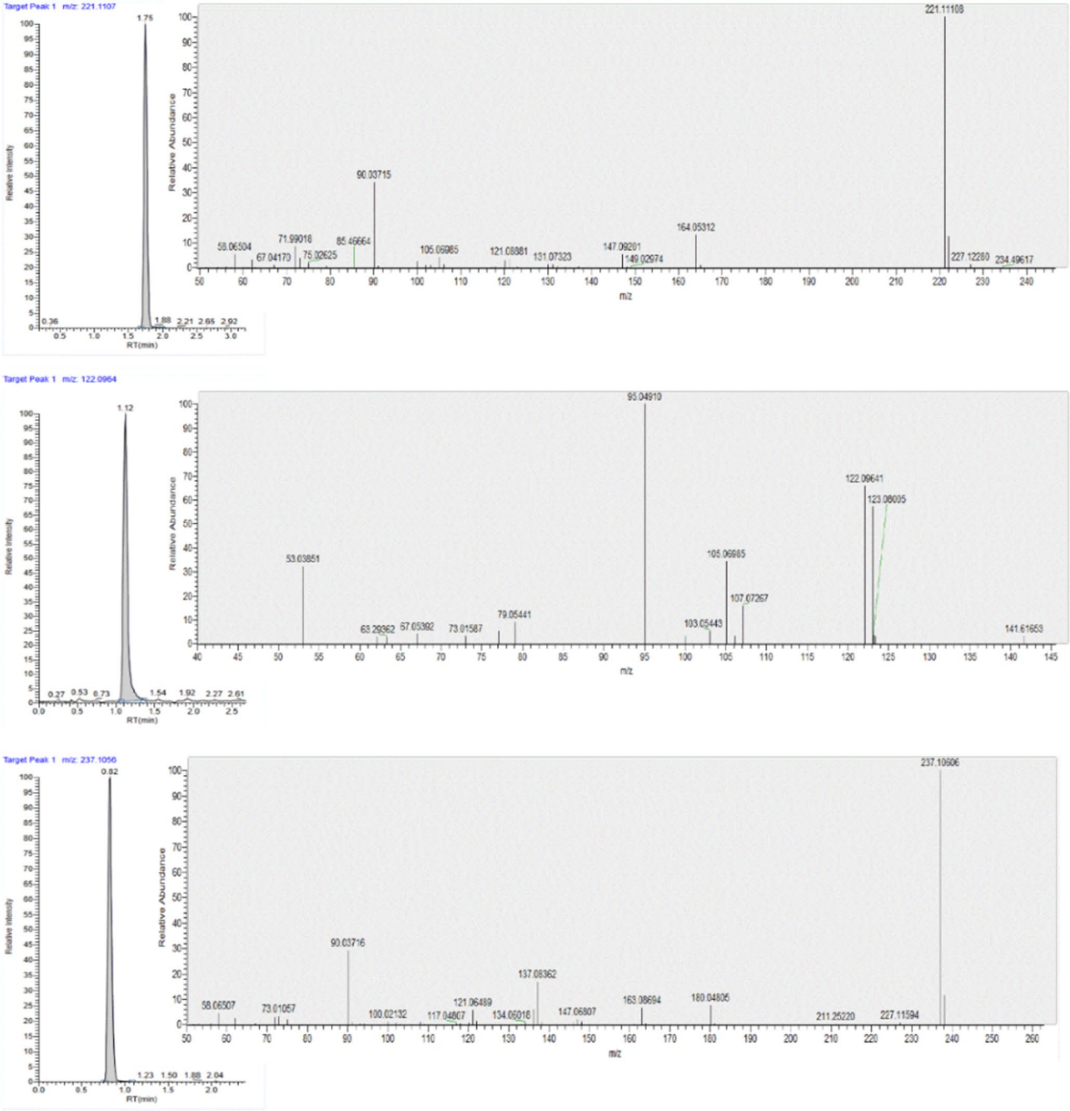
Extracted ion chromatograms and MRM transitions of (a) Xylazine, (b) DMA, and (c) 4-hydroxyxylazine in the spiked sample

Mass spectrometry analysis was performed on an Exploris 120 mass spectrometer coupled with an electrospray ionization probe (Thermo Fisher Scientific, Les Ulis, France) and acquisitions were carried out in multiple reaction monitoring (MRM) mode. The sheath gas flow was 60 arbitrary units (AU), and the auxiliary gas flow rate was set to 15 AU. The vaporization and ion transfer tube temperatures were set to 320 °C and 350 °C, respectively. The ion spray voltage was set to 3400 V and S-lens level was set at 70 eV. Full scan analysis was performed with a mass resolution of 60,000 FWHM within the mass range of m/z 100–350 in positive electrospray ionization mode, followed by a targeted MS2 data dependent acquisition (DDA) analysis with normalized collision energies of 30, 60, and 90% respectively. A mass resolution of 30,000 FWHM and N number of spectra were set at 10 with inclusion list. The isolation window was set to 2 m/z. Raw data was acquired and processed using the Trace Finder Forensic software (v.5.1, Thermo Fisher Scientific, USA). MRM transitions and chromatographic retention times for xylazine, xylazine-d6, DMA, and 4-hydroxyxylazine were optimized with reference standards. The selected transitions and retention times of analytes are reported in Table 1. Additionally, the overlay of chromatograms and MRM transitions of analytes are shown in Fig. 1.

**Table 1:** MRM transitions and retention times of analytes including IS

Analyte	Formula	Precursor Ion (m/z)	Product Ions (m/z)	Rt* (min)
Xylazine	C_12_H_17_N_2_S	221.1107	164.0529, 90.0371	1.73
DMA	C_8_H_12_N	122.0964	107.0729, 105.0698, 95.0491	1.12
4-hydroxyxylazine	C_12_H_17_N_2_SO	327.1056	180.0479, 90.0371, 163.0868	0.81
Xylazine-d6 (IS)	C_12_H_16_N_2S_D_6_	227.1576	164.0529, 90.0371	1.73

*Analytes of interest retention time (Rt)

## Results

### Method validation

Selectivity, carryover, limit of detection (LOD), limit of quantification (LOQ), calibration curve, accuracy, precision, matrix effect, and stability parameters were evaluated. The method was validated according to the European Medicines Agency Guideline for Bioanalytical Method Validation and Scientific Working Group for Forensic Toxicology Guidelines as applicable [28, 29].

Interference of endogenous matrix compounds was checked for the selectivity within the analytes. Eight different lots of drug-free human blood and urine were selected. Blank and analyte-fortified samples at the LOQ levels were analyzed. No interference was detected in the retention times of analytes and IS. No cross contamination was detected in analyzed blank samples after the highest calibration point standard injection in each run (signal < 20% of the low limit of quantitation signal, and < 5% of the IS signal).

The LOD and LOQ were calculated by analyzing six replicates as standards fortified in blank blood and urine from five different sources, yielding a signal-to-noise ratio (S/N) of at least equal or greater than 3 for LOD and an S/N at least 10 for LOQ. The results were summarized in Table 2.

**Table 2:** Analytical limits for xylazine, DMA, and 4-hydroxyxylazine each in blood and urine samples, *n* = 6 (LOD: limit of detection and LOQ: limit of quantification)

Analyte	Matrix	LOD (ng/mL)	LOQ (ng/mL)
Xylazine	Blood	0.025	0.068
	Urine	0.05	0.12
DMA	Blood	0.04	0.11
	Urine	0.06	0.19
4-hydroxyxylazine	Blood	0.025	0.071
	Urine	0.05	0.14

The LOD of xylazine and its metabolites was from 0.025 ng/mL to 0.040 ng/mL for blood samples and it was from 0.05 ng/mL to 0.06 ng/mL for urine samples.

Nine calibration points from different concentration levels including 0.05, 0.1, 0.5, 1, 5, 10, 25, 50 and 100 ng/mL were used for all analytes over the calibration range 0.05–100 ng/mL using the (1/x) linear regression model (x, concentration). The coefficient of determination (R^2^) was exceeded by greater than or equal to 0.9989 for each analyte. The internal standard method was used for the quantification and the ratios of the analyte signal to the internal standard signal against the concentration of the analytes were plotted against the spiked analyte concentrations. The back-calculated concentrations of all calibrator standards were calculated within ± 15% of the nominal value.

Intra-assay precision and accuracy were calculated by analyzing eight replicates of three quality control (QC) concentration levels including low: 0.5 ng/mL, medium: 25 ng/mL, and high: 75 ng/mL on a single day. Inter-assay precision and accuracy were determined by analyzing eight replicates of three QC concentration levels including low: 0.5 ng/mL, medium: 25 ng/mL, and high: 75 ng/mL over six different days. Intra- and inter-assay precisions were expressed as the coefficient of variation (CV) at each QC concentration level and accuracy was calculated in term of bias (%) as the relative deviation from the nominal concentration. All precision and accuracy values at each QC concentration level were within ± 15% of the nomination concentration as shown in Table 3.

**Table 3:** Precision and accuracy of analytes in blood and urine samples (*n* = 8)

Concentration (ng/mL)	Precision (%)				Bias (%)	
	Intra-day		Inter-day			
	Blood	Urine	Blood	Urine	Blood	Urine
Xylazine						
0.5	6.3	5.3	7.1	8.4	4.3	1.6
25	4.9	3.6	5.9	6.9	0.6	1.3
75	2.7	3.2	4.0	5.8	3.2	2.6
DMA						
0.5	7.6	10.1	11.3	11.2	11.7	6.4
25	5.4	8.3	8.6	9.3	5.8	4.8
75	4.3	6.8	7.0	7.6	4.2	4.9
4-hydroxyxylazine						
0.5	4.7	5.6	5.3	6.3	4.6	3.1
25	3.2	3.9	4.6	4.4	2.7	6.3
75	2.6	2.4	3.2	3.8	2.3	1.7

Recovery, matrix effect, and process efficiency were evaluated at each QC concentration level including low: 0.5 ng/mL, medium: 25 ng/mL, and high: 75 ng/mL with six consecutive injections [30]. The recoveries were within the targeted recovery range 72.1–98.4% and 70.3–93.4% at three QC concentration levels for blood and urine, respectively. The matrix effect values were found to vary between 74.9 and 103.9% and between 78.6 and 95.6% at three QC concentration levels for blood and urine, respectively as shown in Table 4 (CV < ± 15%).

**Table 4:** Recovery and matrix effect for xylazine. DMA, and 4-hydroxyxylazine each in blood and urine (*n* = 6)

Concentration (ng/mL)	Recovery (%)		Matrix Effect (%)	
	Blood	Urine	Blood	Urine
Xylazine				
0.5	91.8	86.7	97.1	88.4
25	94.6	84.5	103.9	92.9
75	97.5	87.3	94.0	95.6
DMA				
0.5	74.2	70.3	81.7	78.6
25	72.1	72.8	80.1	82.4
75	73.7	74.6	74.9	79.9
4-hydroxyxylazine				
0.5	97.4	91.2	87.3	90.2
25	95.0	93.4	95.6	88.3
75	98.4	92.5	88.2	84.6

Stability was tested for processed samples at + 8 °C in the autosampler for 72 h, short-term stability at + 4 °C for five days and freeze and thaw stability at −20 °C for five days on blood and urine samples which were spiked with low, medium, high QC concentration levels. The peak areas of the stored samples and freshly prepared samples were compared. Our results showed that they were within the acceptance criteria (± 10%) and all analytes were found to be stable under the conditions studied. Analytical performance data showed that the method was stable and reliable for quantifying xylazine and its two main metabolites.

### Authentic sample analysis

Blood and urine samples of 9,123 cases were investigated for drug abuse between 01 January 2024 and 31 December 2024 by the Chemistry Department of the Forensic Medicine Council of the Ministry of Justice, Ankara. All of the biological samples (blood and urine) examined in this study were obtained from living individuals under the supervision of law enforcement authorities due to suspected substance use (e.g. as part of routine substance abuse testing). No post-mortem or autopsy cases were evaluated. The cases were suspected drug users who were in custody because of drug abuse, illegal drug possession, fighting, robbery, domestic violence, sexual abuse, and rape. For the screening of therapeutic and abused drugs including xylazine, a qualitative LC-HRMS method was performed following a quantitative LC-MS/MS method collaboratively, which could detect approximately 411 drugs with their metabolites including opioids, new psychoactive substances, depressants, and adulterants.

Of the 9,123 cases, 7,581 (83.1%) samples tested positive for one or more drugs of abuse. Methamphetamine (81.3%) was the most abused drug followed by synthetic cannabinoids (73.6%) including MDMB-4en-PINACA, ADB-BUTINACA, ADB-4en-PINACA, MDMB-INACA, ADB-INACA, 4 F-MDMB-BUTINACA, and 5 F-ADB. Cannabis (38.6%), cocaine (22.1%), heroin (14.8%) and MDMA (10.8%) were also found positive in samples of cases, respectively.

The samples of 9,123 cases were analyzed by the validated LC-HRMS method and a total of 28 cases were found positive for xylazine (0.3%). This is the main finding of our study. The mean age of these cases was 28 (18–67) year. Of 28 cases, xylazine was detected in urine samples of 28 (100%) cases (ranging from 0.7 ng/mL to 23.1 ng/mL, mean = 7.8 ng/mL). On the other hand, xylazine was found only in blood samples of 7 (25%) cases (ranging from 0.5 ng/mL to 1.1 ng/mL, mean = 0.7 ng/mL). DMA, which is the major metabolite of xylazine was only detected in 7 (25%) urine samples (detection range 2.8–14.0 ng/mL, mean = 7.9 ng/mL) together with xylazine. Finally, 4-hydroxyxylazine was detected in urine samples of 22 (78%) cases (detection range 0.3-8,2 ng/mL, mean = 2.5 ng/mL). Of 22 cases, 4-hydroxyxylazine was detected in 17 cases only with xylazine, and it was detected in 5 cases with xylazine and DMA.

0f 28 xylazine positive cases, 9 (32%) cases were tested positive for xylazine without an illicit drug, but 19 (68%) cases used polysubstance. The most frequently reported substances used with xylazine were pregabalin which is a GABA analog with anticonvulsant effect in 14 cases (50%) and methamphetamine which is a synthetic stimulant with high addiction potential in 8 (28%) cases. 0f 28 cases, 8 (28%) cases were positive for one additional substance, 8 (28%) for two, and 3 (10%) for three additional substances with xylazine in blood and urine samples. Demographics characteristics of cases, forensic detection of xylazine with its metabolites, and other concomitant drugs were reported in Table 5 (*n* = 28).

**Table 5:** Demographics and forensic characteristics of xylazine positive cases, ankara, 2024 (*n* = 28)

Case number	Sex	Age	Xylazine in blood	Xylazine in urine	Concomitants use of other drugs in blood and urine (not quantified)
1	M	21	Negative	Xylazine (0.7 ng/mL) DMA (2.8 ng/mL)	Blood: Negative Urine: Negative
2	M	18	Negative	Xylazine (2.1 ng/mL)	Blood: Negative Urine: Negative
3	M	31	Negative	Xylazine (5 ng/mL) DMA (14.0 ng/mL) 4-hydroxyxylazine (0.3 ng/mL)	Blood: Paracetamol Urine: Methamphetamine, Amphetamine, Pregabalin, Morphine, Codeine, Paracetamol
4	M	26	Negative	Xylazine (3.6 ng/mL) 4-hydroxyxylazine (0.7 ng/mL)	Blood: Negative Urine: Negative
5	M	26	Negative	Xylazine (4.2 ng/mL) 4-hydroxyxylazine (1.3 ng/mL)	Blood: Negative Urine: Negative
6	M	28	Negative	Xylazine (3.4 ng/mL) DMA (8.6 ng/mL)	Blood: Venlafaxine (24 ng/mL) Urine: Venlafaxine
7	M	46	Xylazine (0.6 ng/mL)	Xylazine (7.0 ng/mL) 4-hydroxyxylazine (2.0 ng/mL)	Blood: Pregabalin (464 ng/mL) Urine: Pregabalin
8	M	67	Xylazine (0.5 ng/mL)	Xylazine (16.1 ng/mL) 4-hydroxyxylazine (1.5 ng/mL)	Blood: Quetiapine (100 ng/mL) Urine: Quetiapine
9	M	18	Negative	Xylazine (11.8 ng/mL) 4-hydroxyxylazine (1.3 ng/mL)	Blood: Negative Urine: Paliperidone
10	M	33	Negative	Xylazine (2.4 ng/mL)	Blood: Negative Urine: Negative
11	M	26	Xylazine (0.6 ng/mL)	Xylazine (14.2 ng/mL) DMA (9.5 ng/mL) 4-hydroxyxylazine (2.4 ng/mL)	Blood: Quetiapine (5 ng/mL) Urine: Quetiapine, Pregabalin
12	M	27	Negative	Xylazine (1.1 ng/mL) 4-hydroxyxylazine (0.6 ng/mL)	Blood: Negative Urine: Morphine, Codeine, Paracetamol
13	M	32	Negative	Xylazine (1.1 ng/mL) 4-hydroxyxylazine (2.1 ng/mL)	Blood: Methamphetamine, Amphetamine, Pregabalin Urine: Methamphetamine, Amphetamine, Pregabalin
14	M	28	Negative	Xylazine (11.0 ng/mL) 4-hydroxyxylazine (0.6 ng/mL)	Blood: Methamphetamine, Amphetamine, Pregabalin Urine: Methamphetamine, Amphetamine, Pregabalin
15	M	32	Xylazine (0.9ng/mL)	Xylazine (17.8 ng/mL) 4-hydroxyxylazine (1.2 ng/mL)	Blood: Methamphetamine, Amphetamine, Pregabalin Urine: Methamphetamine, Amphetamine, Pregabalin
16	F	29	Negative	Xylazine (3.2 ng/mL) DMA (4.7 ng/mL) 4-hydroxyxylazine (2.3 ng/mL)	Blood: Negative Urine: Negative
17	M	46	Xylazine (0.7 ng/mL)	Xylazine (6.7 ng/mL) DMA (7.9 ng/mL) 4-hydroxyxylazine (3.9 ng/mL)	Blood: Negative Urine: Negative
18	M	33	Negative	Xylazine (9 ng/mL) 4-hydroxyxylazine (1.7 ng/mL)	Blood: Methamphetamine, Amphetamine, Pregabalin Urine: Methamphetamine, Amphetamine, Pregabalin
19	M	26	Negative	Xylazine (4.9 ng/mL) 4-hydroxyxylazine (1.3 ng/mL)	Blood: Negative Urine: Methamphetamine, Amphetamine
20	M	21	Negative	Xylazine (9.3 ng/mL) DMA (7.8 ng/mL) 4-hydroxyxylazine (4.9 ng/mL)	Blood: Sertraline Urine: Sertraline, Pregabalin
21	M	23	Xylazine (1.1 ng/mL)	Xylazine (23.1 ng/mL) 4-hydroxyxylazine (8.2 ng/mL)	Blood: Pregabalin Urine: Pregabalin
22	M	30	Negative	Xylazine (4.1 ng/mL)	Blood: Negative Urine: Negative
23	M	28	Negative	Xylazine (14.3 ng/mL) 4-hydroxyxylazine (4.2 ng/mL)	Blood: Risperidone Urine: Risperidone, Paliperidone, Pregabalin
24	M	22	Xylazine (0.8 ng/mL)	Xylazine (11.8 ng/mL) 4-hydroxyxylazine (1.3 ng/mL)	Blood: Negative Urine: Methamphetamine, Amphetamine, Pregabalin
25	M	51	Negative	Xylazine (7.6 ng/mL) 4-hydroxyxylazine (3.1 ng/mL)	Blood: Morphine, Paracetamol Urine: Methamphetamine, Amphetamine, Pregabalin, Morphine, Codeine, Paracetamol, 6-MAM
26	M	33	Negative	Xylazine (1.9 ng/mL)	Blood: Negative Urine: Negative
27	M	26	Negative	Xylazine (8.2 ng/mL) 4-hydroxyxylazine (5.6 ng/mL)	Blood: Pregabalin Urine: Pregabalin
28	M	23	Negative	Xylazine (13.1 ng/mL) 4-hydroxyxylazine (4.3 ng/mL)	Blood: Pregabalin Urine: Pregabalin, Risperidone, Paliperidone

## Discussion

The lack of regulation of xylazine as a controlled substance in both the United States and Europe makes it readily accessible for abuse in human populations. The increased abuse of xylazine became an emergent public legal problem in the United States during the COVID-19 pandemic [31]. One of the major negative effects of globalization is sharing illegal behaviors like drug abuse through social media. For this reason, our society faces the same potential risk, and understanding the prevalence of xylazine abuse is crucial for planning mitigation and response strategies against xylazine-involved non-fatal and fatal overdose cases.

Laboratory diagnosis of xylazine intoxication is still a challenge from the perspective of forensic and legal medicine, especially in cases with polysubstance use [6, 31]. It was reported that widespread implementation of hospital- and community-based xylazine testing capability for biologic samples is crucial for the medical management of opioid overdose cases [7]. Emergency departments which have especially more opioid related overdose cases should be equipped with xylazine test strips for biological specimens as xylazine has a serious impact on clinical course of opioid overdose cases.

The diagnosis of xylazine-involved overdose cases was mostly delayed due to inappropriate toxicological analysis capabilities and improper treatment protocols for polysubstance intoxication [17]. There is no available antidote for xylazine-positive opioid intoxication. The literature shows that combining use of xylazine with opioids could increase the risk of fatal overdose cases. Health professionals should know that sedative effects of xylazine last between 8 and 72 h in non-fatal overdose cases [4–7, 9, 10, 12, 18, 19, 32, 33].

Recent studies are still published about bioanalytical detection of xylazine in human biological samples [10]. Bioanalytical techniques including LC-MS/MS and UHPLC-QTOF are successfully used in forensic toxicological analysis of human biological samples for illicit drug screening because of their high selectivity and sensitivity with reliable results [34]. Whole blood, serum, and urine are the most common samples in humans which are used for the detection of xylazine. However, the mean LOD’s of these studies was generally 0.4 ng/mL for blood samples and 0.3 ng/mL for urine samples [5]. Gao et al. reported a UHPLC-QTOF study for simultaneous determination of xylazine and 2,6-dimethylaniline (DMA) in human blood and urine [24]. The LOD for xylazine and DMA in blood reported 0.2 and 0.1 ng/mL, in urine are 0.4 and 0.2 ng/mL; the LOQ for xylazine and DMAin blood are 0.6 and 0.3 ng/mL, in urine are 1.0 and 0.6 ng/mL, respectively [25]. A recent study reported the first fatal overdose due to tranq-dope in the European Union (Italy) in 2024. In this study, Trana et al. used a validated LC-HRMS method with a LOD of 0.2 ng/mL and LQQ of 1.0 ng/mL for blood and urine samples with 15 min. total tun time [26]. On the other hand, our quantative LC-HRMS method which used SPE detected xylazine in 500 µL sample with a LOD of 0.025 ng/mL for human blood and 0.05 ng/mL for human urine in 7 min. Compared with the reported other bioanalytic chromatographic methods, our LC-HRMS method achieved lower LODs than other reported methods including LC-MS/MS, UHPLC-QTOF, and other LC-HRMS techniques [5, 10, 11, 34]. For this reason, the achieved analytical limit of our study makes our method available for the detection of xylazine and its metabolites in blood and urine samples of suspected cases. Besides, the total run time of our method was shortened by %50 (7 min) when compared with the total run time of the recent LC-HRMS method so our validated LC-HRMS method has the advantage of saving significant amount time for the bioanalysis of xylazine in routine screening tests [11, 35].

It was reported that plasma concentrations of xylazine in non-fatal cases ranged from 0.03 to 4.6 mg/L [10]. However, in our study the highest xylazine concentrations in blood and urine samples were 1.1 ng/mL and 23.1 ng/mL respectively but we could not interpret the values that we found from the perspective of the clinical course in non-fatal or fatal overdose cases because it was highlighted that there was an overlap between non-fatal and fatal cases so there was no safe or toxic dose for xylazine use in humans. Although there are several reported data about lethality of xylazine on rodents, there is not enough data about LD50 of xylazine in humans despite recent studies on its toxicological characteristics because toxic effects of xylazine depend on its combination with other drugs [17]. On the other hand, it was reported that the average dose of xylazine ranged between 40 and 2.400 mg in fatal cases and dose-dependent increases in lethality due to xylazine were observed within 0.5 log units [6, 10, 12, 15, 16, 18]. Ayub et al. reviewed 34 studies of 59 cases that used xylazine and they presented the data including concomitant use of other drugs. They reported that 28 cases used fentanyl, morphine, heroin, codeine, alcohol, and cocaine in combination with xylazine [6]. In our study, we found that the most frequently reported substances used with xylazine were pregabalin (50%) and methamphetamine (28%), respectively. A rather large number of cases (9123) in 2024 have been analyzed, with 7581 tested positive for drugs of abuse, amongst these methamphetamine and interestingly synthetic cannabinoids were the most relevant drugs detected. Numbers for common drugs of abuse were rather low in comparison to these findings. For Xylazine, 0.3% (28) of the cases were positive, all of them (28) in urine and 25% (7) of them in blood samples. In 9 cases (one third of all xylazine positive cases) xylazine was the only detected drug. The combination with pregabaline was found most frequently.

These findings suggest a noteworthy trend in the use of illicit drugs (or drugs with abuse potential, such as xylazine and pregabalin) in Turkey, which appears to differ from patterns observed in other countries, including the USA and European nations. As shown in Table 5, xylazine was detected in combination with opiates in only two cases, and notably, no cases involved its combination with cocaine. Importantly, there were no instances of xylazine being used in a cocaine/heroin mixture (commonly referred to as “speedball”) or in combination with fentanyl—patterns that are well-documented and highly relevant in the US context. This distinction should be emphasized when interpreting regional differences in drug use trends and associated risks.

Pregabalin is a GABA analogue, and it is used for the treatment of epilepsy, neuropathic pain, generalized anxiety disorder, fibromyalgia, postherpetic neuralgia, and neuropathic pain following spinal cord injury or diabetes mellitus. However, case reports noted that abuse potential of pregabalin appears at higher dosages for becoming “high” or potentiating the effect of opioids. Grosshans et al. suggested that pregabalin had potential for abuse among patients with past or current opiate intake [36, 37]. In animal studies, pregabalin administration was used for premedication before xylazine for standing sedation in horses [38]. Pregabalin was found in 14 (50%) of our cases so we hypothesized that the abuse of pregabalin at higher dosages in combination with xylazine could be a recent emerging threat which could cause a new fatal polydrug overdose type.

Rock et al. emphasized that xylazine was found frequently with fentanyl, cocaine, heroin, and benzodiazepines in biological samples of overdose cases in North America and they reported the first xylazine-involved fatal opioid overdose case in the United Kingdom and Europe [18]. We found similar illicit drugs like methamphetamine and heroin in our cases respectively, and we think that the rising burden of xylazine presence in biological samples of our cases reflects the current production trend on adherent of illicit drugs in the drug supply all over the world.

Kacinko et al. screened approximately 170,000 cases between January 2019 and June 2021. While the xylazine positivity rate in cases was 0.8% in January 2019, it was 3.6% in June 2021 with a 4-fold increase 2.5 year [20]. In our study, we found the xylazine positivity in Ankara during 2024 as 0.003%. Like Rock et al. and Malaca et al., our findings also suggest that the presence of xylazine and its metabolites in drug users should be screened in standard toxicological analysis panels of forensic laboratories to estimate the composition of illicit opioid drugs in the drug supply and to determine the prevalence of potential xylazine-involved overdose cases [12, 14, 18].

Finally, we noted that only xylazine itself was identified in blood samples in 7 (25%) cases because of its relatively short plasma half-life (4.4 h) which complicates its metabolites detection in blood samples after a prolonged period. We found that blood xylazine concentrations were less than 5% of urine xylazine concentration in most cases so we concluded that using urine samples is more advantageous for drug screening due to the greater ease of detection of both parent compounds and their metabolites over an extended time compared to blood for xylazine screening. Also, while DMA was detected in urine samples of 7 (25%) xylazine positive cases, 4-hydroxyxylazine was detected in urine samples of 22 (78%) xylazine positive cases. We hypothesized that 4-hydroxyxylazine might be evaluated as the target metabolite of xylazine in forensic screening methods.

Limitations of our study require some consideration. First, we analyzed only the biological specimens of suspected illicit drug users from Ankara region during 2024 and there are other analysis laboratories of Council of Forensic Medicine all around Türkiye. For this reason, we do not have enough data for the estimation of national prevalence of xylazine abuse, so the numbers reflect probably a gross underestimate of the true number of our society. Secondly, we had no data about the reason of preference about using xylazine alone or with other illicit drugs and the route of xylazine use. Also, we did not know whether illicit use of xylazine was either a concordant or a discordant behavior. Like Copeland et al., we also think that xylazine could have been used as an adulterant in methamphetamine and heroin formulations or it could have been sold to drug users as a counterfeit tablet [39]. Confiscated samples—particularly those involving methamphetamine, heroin and pregabalin—were analyzed by the Narcotics Department of the Turkish National Police, the Ministry of Trade’s Customs Enforcement, and the Narcotics Department of the Council of Forensic Medicine. However, it should be noted that not all substances circulating in the illicit drug market were subject to investigation. To date, there is no evidence indicating the use of xylazine as an adulterant in these substances.

To our best knowledge, this is the first study from Türkiye which reports illicit use of xylazine in Ankara during 2024 by using a validated and fast LC-HRMS method and it shows the entry of xylazine to the local drug supply. Future studies should focus on the prevalence of xylazine abuse in the whole country.

## Conclusion

Xylazine abuse is a widespread emerging threat; therefore, understanding its metabolism and developing effective screening techniques for xylazine and its metabolites in blood and urine are crucial for forensic and clinical toxicology. We developed and validated a fast, sensitive, and practical bioanalytical LC-HRMS method for the determination of xylazine, and its corresponding metabolites in human biological samples including blood and urine. By using this method, we reported on the first detection of xylazine in samples of suspected drug users in Ankara during 2024. As an adherent of most illicit drugs, the prevalence of xylazine abuse is an important clinical concern which could increase the frequency of overdose cases and deaths. Our findings highlight the need to monitor the prevalence of xylazine use in the illicit drug market on a national scale to design harm reduction strategies aimed at mitigating illicit xylazine abuse before it escalates as an emerging threat.

## Data Availability

The datasets used in the current study are available from the corresponding author upon reasonable request.
